# Changing epidemiology and trends in incidence of kidney cancer in England, 1985-2019

**DOI:** 10.1093/eurpub/ckac130.052

**Published:** 2022-10-25

**Authors:** Y Salari, P Bannister, A Memon

**Affiliations:** Brighton and Sussex Medical School, Department of Primary Care and Public Health, Brighton, UK

## Abstract

**Background:**

Kidney cancer is the 7th most common cancer in the UK, accounting for 4% of all new cancer cases. The risk factors for kidney cancer include obesity, smoking, hypertension, and exposure to certain environmental and occupational carcinogens. We conducted a retrospective population-based cohort study to examine whether there have been changes in the incidence of kidney cancer in England during the past four decades.

**Methods:**

Individual level data for patients diagnosed with kidney cancer in England during 1985-2019 were obtained from the Office for National Statistics/Public Health England. Average annual incidence rates were calculated by two age categories (0-49, 50+ years) and all ages combined during the seven five-year time periods (1985-89 to 2015-19). The percentage change in incidence was calculated as change in the average annual incidence rate from the first (1985-89) to the last time period (2015-19).

**Results:**

During the 35-year study period, a total of 197,819 new cases of kidney cancer were registered in England (62.4% males, 37.6% females). In young people aged 0-49 years, the average annual incidence rates increased by 164% in males and 144% in females (from 1.4/100,000 in 1985-89 to 3.7/100,000 in 2015-19 in males and from 0.9/100,000 in 1985-89 to 2.2/100,000 in 2015-19 in females). In older people aged 50+ years, the rates increased by 129% in males and 147% in females (from 24.5/100,000 in 1985-89 to 56.0/100,000 in 2015-19 in males and from 11.9/100,000 in 1985-89 to 29.4/100,000 in 2015-19 in females).

**Conclusions:**

There has been a steady and significant increase in the incidence of kidney cancer in England over the past four decades. The largest increase (164%) was observed in young males aged 0-49 years, which was unexpected. Considering the risk factors for kidney cancer, further research is needed to understand the role of environmental/occupational exposures in causing kidney cancer.

**Key messages:**

- About 24% of kidney cancers in the UK are attributed to obesity and 13% to smoking. This research highlights the benefits of reducing the prevalence of obesity and smoking in the general population.
- The unexpected significant increase in the incidence of kidney cancer in young people aged 0-49 years needs further investigation.

